# Humoral Immune Abnormalities in Transient Childhood Neutropenia: Insights From a 10-year Cohort Study in a Tertiary Center

**DOI:** 10.1007/s10875-026-02042-w

**Published:** 2026-06-18

**Authors:** Emmanuele Schorn, Maarja Soomann, Seraina Prader, Jana Pachlopnik Schmid, Johannes Trück

**Affiliations:** Division of Immunology and the Children’s Research Center, University Children’s Hospital Zurich, University of Zurich, Lenggstrasse 30, Zurich, 8008 Switzerland

## Abstract

**Purpose:**

Transient neutropenia in early childhood is a relatively common condition often associated with neutrophil-specific autoantibodies; however, its connection to broader humoral immune system abnormalities remains poorly understood.

**Methods:**

The current study investigated this relationship through a retrospective cohort analysis at a pediatric tertiary center in Switzerland.

**Results:**

In total, 92 children aged 0 to 6 years seen at our hospital between January 2014 and December 2023 were included in the final cohort. Of the 68 children who underwent detailed immunological testing, 52 (77%) exhibited humoral abnormalities, with 24 (35%) classified as moderate and 28 (41%) as mild. Patients with humoral abnormalities exhibited neutrophil-specific autoantibodies less commonly (56% vs. 100%, *p* = 0.003), had higher minimum absolute neutrophil counts (ANC) (median minimum ANC 0.26 vs. 0.05 × 10^9^/L, *p* = 0.002), and a shorter duration of neutropenia (median duration 12.1 vs. 28.5 months, *p* = 0.001) compared to patients without abnormalities. Additionally, 36% of patients with humoral abnormalities had inadequate vaccine antibodies, compared to none in the group without abnormalities. These findings suggest that patients with humoral abnormalities may have a higher risk of infection and could benefit from additional counseling and timely booster vaccinations.

**Conclusion:**

This study highlights a potential causal relationship between humoral immune system abnormalities and transient neutropenia in early childhood. Routine immunological assessments in children with early-onset (autoimmune) neutropenia are likely to aid in patient management and family counseling.

**Supplementary Information:**

The online version contains supplementary material available at https://doi.org/10.1007/s10875-026-02042-w.

## Introduction

Despite its frequency, transient neutropenia in early childhood remains poorly understood, with numerous empirical observations of associated humoral immune system abnormalities that have yet to be systematically investigated. This condition includes autoimmune neutropenia (AIN) and idiopathic neutropenia (IN), with AIN being the most common cause of neutropenia in children, occurring at an annual incidence of approximately 1 in 100,000 [1]. Notably, neutropenia is often detected incidentally, with more than one in four cases identified by chance [2]. Irrespective of the severity of neutropenia, patients with AIN typically exhibit only mild infectious symptoms [3–5], and spontaneous remission occurs in 95% of patients, usually within two to three years [3].

AIN is believed to have a complex and multifactorial etiology involving several key mechanisms. Neutrophil-specific autoantibodies, particularly those targeting HNA-1a, HNA-1b and pan-FcγRIIIb, play a significant role in neutrophil destruction, with different antibodies implicated in primary and secondary AIN [6, 7]. Additionally, genetic predispositions may contribute, as variants in genes related to immune regulation have been associated with long-lasting and late-onset AIN [8–10]. Abnormalities in regulatory T cells, including decreased frequency and reduced expression of key molecules, have also been correlated with AIN [11, 12]. Other contributing factors include molecular mimicry from microbial antigens [12] and bystander activation of autoreactive B cells [13].

The observed prevalence of (secondary) AIN in 1–3% of patients with common variable immunodeficiency (CVID) [14, 15] is significantly higher than the incidence of 1 in 100,000 in the general population [1], supporting the hypothesis of a link between humoral immune system abnormalities and AIN. Previous studies have primarily focused on the prevalence of autoimmune cytopenia in patients with CVID [14–20], leaving the relationship between isolated, early onset transient autoimmune neutropenia, and humoral immune system abnormalities largely unexplored. Furthermore, comprehensive data on transient neutropenia in early childhood are generally scarce, with most available information derived from a single Italian registry [21].

The clinical management of transient neutropenia in early childhood is complicated by the low sensitivity, specificity, and availability of autoantibody testing, which otherwise aids patient management, prognosis, and diagnostics. However, previous studies have suggested that patients with and without positive neutrophil-specific autoantibodies exhibit the same phenotype [22]. To further explore the relationship between transient neutropenia and abnormalities of the humoral immune system, we conducted a retrospective cohort study analyzing data from all children meeting the clinical presentation of AIN at our institution over a 10-year period.

## Methods

### Study Design and Patient Cohort

We conducted a retrospective, single-center, cohort study using data extracted from hospital records at the University Children’s Hospital of Zurich, covering the period from January 1, 2014 to December 31, 2023. The study was approved by the ethics committee of the Canton of Zurich (BASEC no. 2023 − 00788). Inclusion required general consent permitting retrospective use of clinical records. Where consent status was unclear, parents or legal guardians were contacted by telephone to document consent as required by the ethics committee. Patients who declined consent or whose consent could not be confirmed were excluded. Most patients were managed under the Division of Immunology, with the remainder managed by the Divisions of Hematology or Infectious Diseases.

### Inclusion and Exclusion Criteria

The inclusion criteria were: (1) age 0 to 6 years at the time of neutropenia onset, and (2) neutropenia lasting for at least 6 weeks. Exclusion criteria were: (1) an identifiable cause of neutropenia other than autoimmunity (e.g., constitutional neutropenia, alloimmune neutropenia, neutropenia associated with myeloproliferative disorders, neutropenia related to acquired bone marrow failure, medication-induced neutropenia, and infection-associated neutropenia as proposed by Fioredda et al. [23]), or (2) remission before 4 months of age.

### Definitions

AIN was defined as isolated neutropenia lasting more than 6 weeks with positive neutrophil-specific autoantibodies and no other identifiable cause, as per the criteria proposed by Fioredda et al. 2022 [23]. IN was defined as neutropenia lasting more than 6 weeks, with either negative or no autoantibody testing and no other identifiable cause. Infections were classified as mild if documented without hospitalization and as severe if requiring hospitalization. Humoral abnormalities were defined as any of the following: (1) quantitative immunoglobulin deviations below age-adjusted reference ranges (IgG, IgA, IgM; and, where available, IgG subclasses), (2) abnormalities in B-cell subsets on flow cytometry using age-adjusted reference values, and/or (3) inadequate vaccine-specific antibody responses. For statistical analyses, humoral abnormalities were categorized pragmatically (moderate, mild, none) to describe immune deviation without assigning a formal antibody deficiency entity. Moderate abnormalities were defined as IgG deficiency and/or inadequate vaccine antibody levels, while mild abnormalities were defined as isolated IgA and/or IgM deficiency, IgG subclass deficiency, and/or abnormalities in B-cell differentiation, in the absence of moderate abnormalities. Table S1 provides the definitions utilized throughout this paper.

### Data Collection and Extraction

**Fig. 1:**
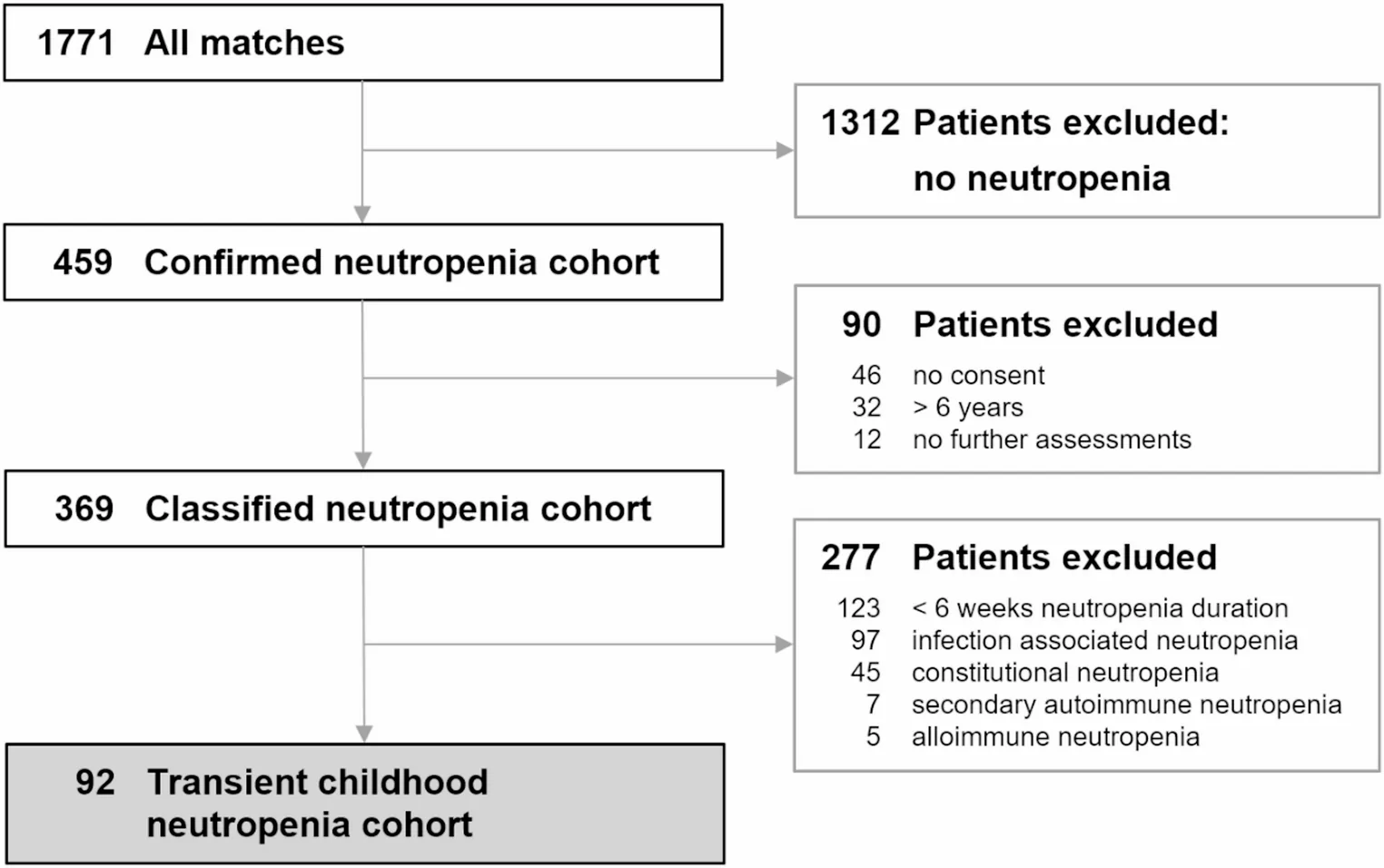
Patient flow during enrolment. Detailed exclusion criteria in each step are presented in Table S3

Eligible patients were identified through a hospital-wide electronic medical record search for cases of neutropenia during the specified study period, independent of treating department or reason for presentation (search terms: Table S2). A sequential filtering process was conducted, starting with the exclusion of all non-neutropenic cases. The remaining patient records were manually reviewed and classified into predefined neutropenia categories (Table S3), enabling identification of individuals with AIN and IN to establish the final study cohort (Fig. 1). Patient histories and immunological data were extracted from electronic records. Vaccine-specific antibody concentrations and flow cytometry results were interpreted by two investigators in the context of documented vaccination history, time since the most recent dose, and prematurity status, using age-appropriate protection thresholds. Patients with insufficient vaccination documentation were excluded from vaccine-response analyses. Based on the definitions above, patients were classified into moderate, mild, or no humoral abnormalities.

### Laboratory Evaluation and Follow-Up

Comprehensive immunological data, including differential blood counts, immunoglobulins (IgG, IgA, IgM, partially also IgG subclasses), vaccine-specific antibodies (primarily tetanus toxoid, *Haemophilus influenzae* type b, and pneumococcal polysaccharide antibodies), and flow cytometry lymphocyte phenotyping were available for most patients. Follow-up was typically quarterly or semiannual, but laboratory assessments performed and overall follow-up schedules varied by specialty, physician practice, patient age and other patient-specific factors. Upon confirmation of neutropenia, neutrophil-specific autoantibodies were generally assessed alongside comprehensive testing, often following published diagnostic algorithms [23, 24], although testing was often omitted at higher neutrophil counts due to presumed reduced assay sensitivity in such cases [2]. In selected patients, particularly those with baseline negative neutrophil-specific autoantibodies or features suggesting alternative etiologies for neutropenia, genetic analyses and/or bone marrow aspirates were performed. Recovery was defined as a documented normalized ANC with no subsequent neutropenic values and led to discharge from further monitoring. Neutrophil-specific autoantibody testing was performed at the DRK Blutspendedienst West (Germany) using internationally standardized methods, including the Granulocyte Immunofluorescence Test (GIFT), Granulocyte Agglutination Test (GAT), and Monoclonal Antibody Immobilization of Granulocyte Antigens (MAIGA) [13, 25, 26].

### Statistical Analysis

All statistical analyses were conducted using R (version 4.3.0; packages listed in Table S4) [27, 28]. Continuous variables are reported as median and range and categorical variables as frequencies and percentages. Group comparisons were performed using the Mann-Whitney U and the Kruskal-Wallis test for continuous variables and the chi-square test or Fisher’s exact test for categorical variables, depending on group size. The association between white blood cell count (WBC) and ANC was assessed using Spearman’s rank correlation overall and within neutropenic and leukopenic ranges (WBC < 4.0 × 10^9^/L). Time to remission was assessed using time-to-event analysis with the Kaplan-Meier estimator. The log-rank test was used to compare time to remission across patient groups. A sensitivity analysis compared preterm and term patients with respect to key clinical outcomes and laboratory parameters given that neutropenia in preterm infants may represent a distinct entity [29]. Patients without remission or lost to follow-up were right censored at the last recorded follow-up. Statistical significance was set at *p* < 0.05.

## Results

### Demographic and Clinical Characteristics

**Fig. 2:**
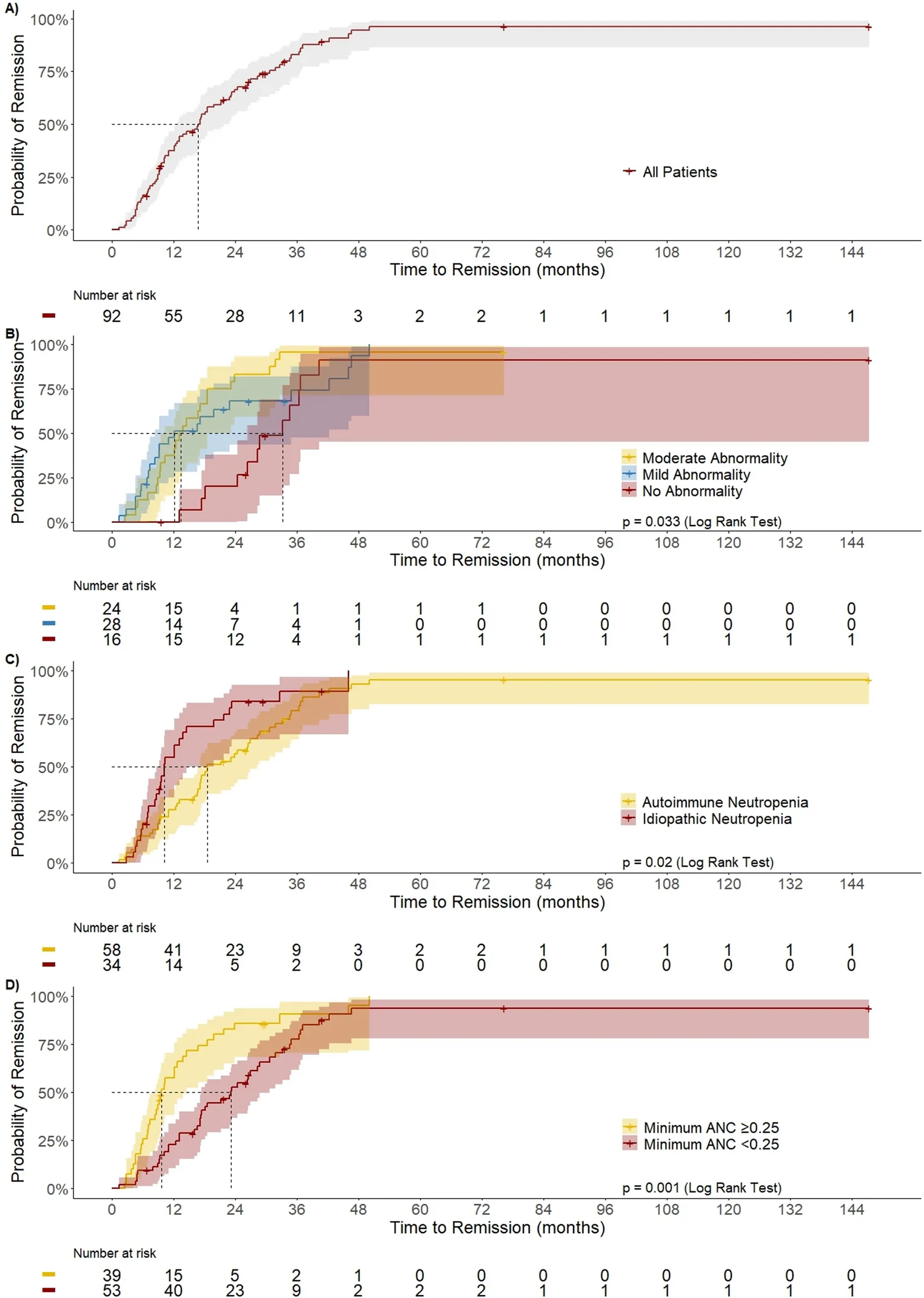
Time to remission of neutropenia. Probability of remission including 95% confidence interval is shown for **A** all patients, **B** patients with moderate, mild and no humoral abnormalities, **C** patients with and without positive neutrophil-specific autoantibodies, and **D** patients with minimum ANC ≥ 0.25 × 10^9^/L and < 0.25 × 10^9^/L. Dashed lines indicate the median time to remission in subgroups

Over the 10-year study period, 459 patients with neutropenia were identified. After sequential filtering and exclusion of patients (Fig. 1 and Table S3), 92 patients remained in the final study cohort. Of these, eight patients were lost to follow-up and five patients remained neutropenic at the end of the study. One patient had late-onset neutropenia (diagnosed after 36 months of age), and nine patients experienced long-lasting neutropenia (persisting for more than 36 months) [8], seven of whom were followed until remission. The distribution of demographic and clinical parameters is presented in Table 1, whereas Fig. 2A shows the time to remission curve for all patients. A subset of 22 patients was born preterm. In a sensitivity analysis, preterm and term patients did not differ significantly in key clinical outcomes and laboratory parameters (Table S5).

**Table 1:** Demographic, clinical and diagnostic characteristics of the study cohort

	All Patients (*n* = 92)	
	Number	(%)
Sex, male	57	(62)
Preterm	22	(24)
Extremely preterm (< 28 weeks)	8	(9)
Very preterm (28–31 weeks)	4	(4)
Moderate to late preterm (32–36 weeks)	10	(11)
Born at term	55	(60)
Unknown gestational age	15	(16)
Context of the initial diagnosis of neutropenia		
Incidental finding^*^	44	(48)
Mild infection^#^	21	(23)
Severe infection^$^	27	(29)
	Median	Range
Age at onset (months)	7.2	(0–36.9)
Age at remission (months)	23.0	(4.1–68.8)
Duration of neutropenia (months)^€^	13.2	(1.3–50.1)

^*^full blood count (FBC) performed for reasons unrelated to infection

^#^FBC due to infection, not hospitalized

^$^FBC due to infection, hospitalized

^€^in those who achieved remission

Granulocyte colony-stimulating factor (G-CSF) was administered to six patients between 2011 and 2020, primarily for recurrent/severe infections or perioperative management. Antimicrobial prophylaxis was administrated to 11 patients between 2012 and 2022, with three of these patients receiving both G-CSF and antimicrobial prophylaxis.

In the whole cohort, a total of 46 infection-related hospitalizations were recorded prior to first documentation of neutropenia, and 87 infection-related hospitalizations were recorded during the neutropenic phase. Before neutropenia was first documented, 67/92 patients (73%) had no mild infections and 25/92 (27%) had one or more mild infection; 55/92 (60%) had no severe infections and 37/92 (40%) had one or more severe infection. During the neutropenic phase, 23/92 patients (25%) had no mild infections and 69/92 (75%) had one or more mild infection; 56/92 (61%) had no severe infections and 36/92 (39%) had one or more severe infection. (Table S6). Respiratory infections were the most common, with 12 cases recorded prior to neutropenia and 44 during the neutropenic phase. Notably, antibiotic-treated respiratory infections increased disproportionately following neutropenia onset (2 cases before vs. 21 cases during neutropenia). Specific infections of interest included sepsis (2 cases before vs. 3 during neutropenia), lymphadenitis (6 cases before vs. 0 during neutropenia), and skin/soft tissue infections (2 cases before vs. 5 during neutropenia). Nine patients experienced more than two severe infections during neutropenia, all of whom had a minimum documented ANC ≤ 0.05 × 10^9^/L. For a detailed breakdown of documented infections, see Table S7.

### Immunological Testing

Immunological testing was performed in 68 of 92 patients (74%), revealing humoral immune system abnormalities in 52, 77% of those tested. All immunoglobulin concentrations and B-cell subset values were interpreted against established, age-adjusted reference ranges. For immunoglobulins, local normative data derived from a healthy pediatric reference cohort at our centre were used; these have been shown to be concordant with manufacturer reference values, supporting the validity of the reported findings. Detailed information on the tests conducted, patient distribution, and results can be found in Table 2. There were no significant demographic and clinical differences between patients who underwent immunological testing and those who did not (comparison of patients with and without immunological testing: Table S8). Moderate humoral immune abnormalities were identified in 24 patients (35%), including 14 patients (21%) with IgG deficiency and 18 patients (27%) with inadequate vaccine antibodies. Mild abnormalities were found in 28 patients (41%), including 17 patients (25%) with IgA and/or IgM deficiency, 2 patients (3%) with IgG subclass deficiency, and 15 patients (22%) with B-cell subpopulation imbalances, indicating disturbed B-cell differentiation (Table 2). 16 patients (23%) had no humoral abnormalities. Among patients undergoing B-cell immunophenotyping, abnormalities in B-cell subpopulations were identified in 39% (Table S9). While the majority of these abnormalities were mild and isolated, they represented consistent deviations from age-adjusted reference ranges across the cohort. B-cell subset abnormalities predominantly consisted of reduced proportions of differentiated subsets, and immunoglobulin reductions, while partial, were observed at a frequency and distribution exceeding expected physiological variation in this age group. Genetic testing and bone marrow biopsy were unremarkable in all tested patients, except for one with long-lasting autoimmune neutropenia, whose bone marrow biopsy demonstrated normal myeloblast numbers but nonspecific decreased myeloid maturation while under G-CSF, with persisting neutropenia at the last follow-up.

**Table 2:** Immunological abnormalities by diagnostic test

Diagnostics	All patients (*n* = 92)	
	No. tested	No. with positive test/abnormalities (%)^*^
Autoantibody-testing	79	58 (73)
GIFT	76	57 (75)
GAT	76	31 (41)
ELISA (MAIGA)	72	35 (49)
Genetic testing	19	0 (0)
Bone marrow examination	10	1 (10)
Antibody testing^#^		
IgG^$^	68	14 (21)
IgA and/or IgM^€^	68	36 (53)
IgG subclasses^€^	19	6 (32)
Vaccine antibodies^$^	68	18 (27)
Flow cytometry		
Total B, T, and NK cell count	70	4 (6)
T-cell subpopulations	69	7 (10)
B-cell subpopulations^€^	61	24 (39)

^*^percentage of patients with abnormalities of all patients tested with the respective test

^**#**^only decreased levels were considered abnormal

^$^Moderate humoral abnormalities (total n = 24)

^€^Mild humoral abnormalities (total n = 28)

*GIFT* granulocyte immunofluorescence test, *GAT* granulocyte agglutination test, *ELISA* enzyme-linked immunosorbent assay, *MAIGA* monoclonal antibody immobilization of granulocyte-specific antigens, No., number

Patients with any form of humoral abnormality achieved neutropenia remission significantly faster than those without (*p* = 0.033, log-rank test, Fig. 2B). Additionally, patients with humoral abnormalities were younger compared to those without abnormalities at both onset of neutropenia (median age 6.2 vs. 12 months, *p* < 0.001) and at remission (median age 19.9 vs. 45.2 months, *p* < 0.001). The context of initial diagnosis of neutropenia varied, with patients exhibiting humoral abnormalities more frequently being found incidentally (50% vs. 13%, *p* = 0.023, Table S10). No significant differences were observed in the demographic, clinical, or diagnostic characteristics between patients with moderate and mild humoral immune abnormalities (Table 3).

**Table 3:** Demographic, clinical and diagnostic characteristics by severity of humoral immune system abnormalities

	Humoral immune system abnormalities			
	Moderate (*n* = 24)	Mild (*n* = 28)	None (*n* = 16)	*p*-value
	Number (%)	Number (%)	Number (%)	
Autoimmune Neutropenia	15 (63)	14 (50)	16 (100)	***0.003***
Idiopathic Neutropenia	9 (37)	14 (50)	0 (0)	
Sex (male)	14 (58)	17 (61)	7 (44)	*0.528*
Preterm	7 (29)	7 (25)	1 (6)	*0.225*
Extremely preterm (< 28 weeks)	4 (17)	1 (4)	0 (0)	
Very preterm (28–31 weeks)	1 (4)	2 (7)	0 (0)	
Moderate to late preterm (32–36 weeks)	2 (8)	4 (14)	1 (6)	
Born at term	14 (58)	17 (61)	12 (75)	
Unknown gestational age	3 (13)	4 (14)	3 (19)	
Context of the initial diagnosis of neutropenia				*0.088*
Incidental finding^*^	12 (50)	14 (50)	2 (13)	
Mild infection^#^	6 (25)	5 (18)	6 (38)	
Severe infection^$^	6 (25)	9 (32)	8 (50)	
	Median (Range)	Median (Range)	Median (Range)	
Age at onset (months)	5.6 (0.8–36.9)	7.0 (0–24.4)	12.0 (5.3–34.2)	***0.002***
Age at remission (months)	20.1 (6.2–68.8)	19.6 (4.1–64.3)	45.2 (18.5–62.9)	***0.001***
Duration of neutropenia (months)^€^	13.1 (2.5–32.6)	9.2 (1.3–50.1)	28.5 (13.2–40.4)	

^*^full blood count (FBC) performed for reasons unrelated to infection

^#^FBC due to infection, not hospitalized

^$^FBC due to infection, hospitalized

^€^ in those who achieved remission

### Neutrophil-Specific Autoantibodies

In our cohort, 96 neutrophil-specific autoantibody tests were conducted, yielding 63 positive (66%) results. Testing was performed once in 64 patients, twice in 12, and three times in 3, while 13 patients underwent no autoantibody testing. Within the IN subgroup (*n* = 34), autoantibody testing was performed once in 15 patients and twice in 6 patients, whereas 13 patients had no autoantibody testing. The median ANC at time of testing was 0.22 × 10^9^/L (range: 0.04 to 2.7 × 10^9^/L) for positive test results and 0.54 × 10^9^/L (range: 0.03 to 1.76 × 10^9^/L) for negative results. Lower ANC values at the time of testing were significantly associated with a higher likelihood of a positive test result (*p* = 0.001). The fraction of positive results was 85% for ANC < 0.25 × 10^9^/L, 68% for ANC 0.25–0.5 × 10^9^/L and 43% for ANC > 0.5 × 10^9^/L.

Patients were categorized into AIN (58 patients) and IN (34 patients) subgroups based on neutrophil-specific autoantibody results (see Table 2 for detailed results). Significant demographic and clinical differences between these subgroups were observed, including age at onset (9 vs. 4.3 months, *p* = 0.001), age at remission (29.2 vs. 16.3 months, *p* = 0.005) and duration of neutropenia (*p* = 0.020, log-rank test), as detailed in Fig. 2C and Table S11. Context of initial diagnosis between AIN and IN differed significantly (*p* = 0.002), with IN being more frequently detected incidentally and AIN being more frequently identified during severe infections. The AIN subgroup included one patient with late-onset neutropenia, seven with long-lasting neutropenia and three who remained neutropenic at last follow-up. The IN subgroup included one patient with long-lasting neutropenia, one who remained neutropenic at follow-up and four patients with infection exposure (2 with congenital HIV exposure and 2 with CMV infection). A comparison between IN patients with (62%) and without (38%) neutrophil-specific autoantibody testing showed no significant differences in demographic, clinical, or diagnostic characteristics, nor in time to remission (Table S12, Figure S1).

Table 3 highlights the disproportionate distribution of AIN and IN subgroups across moderate, mild and no humoral abnormalities. All IN patients who underwent immunological testing were found to have humoral abnormalities (more detail in Table S9). Conversely, neutrophil-specific autoantibodies were detected in all patients without humoral immune abnormalities.

### Neutrophil Counts

In the cohort, 78 patients (85%) had severe neutropenia (minimum ANC < 0.5 × 10^9^/L), while 14 patients (15%) had moderate neutropenia (minimum ANC 0.5–1 × 10^9^/L) [30]. Time to remission did not differ significantly between patients with severe and moderate neutropenia (*p* = 0.311; Figure S2). However, when categorized by minimum ANC ≥ 0.25 × 10^9^/L and < 0.25 × 10^9^/L, a significant difference in remission times was observed (*p* = 0.001, log-rank test; Fig. 2D). Table S13 highlights significant differences in age at onset and remission (*p* < 0.001), with the lower minimum ANC group being older in both characteristics. Context of initial diagnosis between the lower and higher minimum ANC groups differed significantly (*p* = 0.007), with the higher minimum ANC group being more frequently detected incidentally and the lower minimum ANC group being more frequently identified during severe infections.

**Fig. 3:**
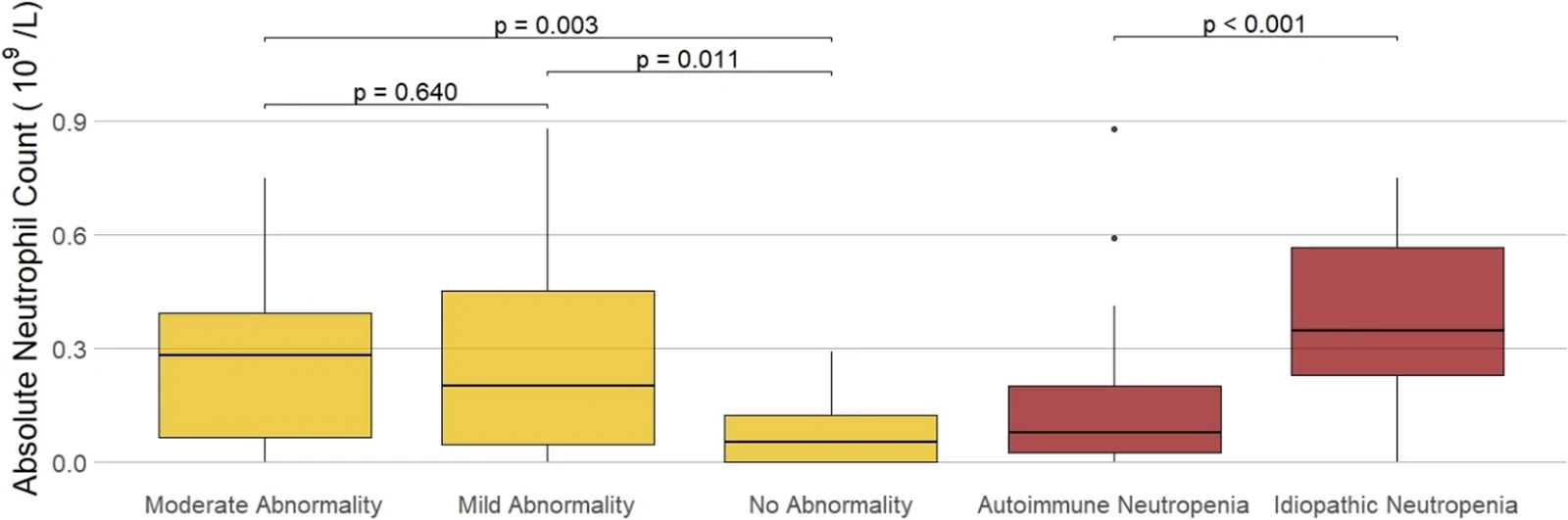
Distribution of minimum absolute neutrophil count across the subgroups moderate, mild and no humoral abnormalities, autoimmune neutropenia and idiopathic neutropenia

The median minimum ANC across the study cohort was 0.14 × 10^9^/L (range 0–0.88 × 10^9^/L). The distribution of minimum ANC values in different subgroups is shown in Fig. 3. Leukopenia was documented in 29/92 patients in at least one complete blood count. The association between WBC and ANC was weak when restricting to neutropenic measurements (Spearman’s ρ = 0.29, *p* < 0.001) or leukopenic measurements (ρ = 0.24, *p* = 0.002).

Over time, patients without humoral abnormalities had lower ANCs during the neutropenic phase but ultimately recovered comparably to those with abnormalities (Figure S3). Additionally, patients who were not tested for humoral immunity recovered from neutropenia at a younger age, with all patient groups converging by the end of the observation period.

## Discussion

This 10-year retrospective study of immunological diagnostics in early childhood transient neutropenia revealed that 77% of tested patients exhibited humoral immune system abnormalities, which were significantly associated with their clinical and laboratory course. Patients with humoral abnormalities had a shorter duration of neutropenia, higher minimum ANC, and lower rates of neutrophil-specific autoantibody detection compared to those without abnormalities. No significant differences were observed between patients with moderate versus mild humoral abnormalities. AIN patients had a longer duration of neutropenia and lower minimum ANC than IN patients, all of whom exhibited some degree of humoral abnormality. Higher minimum ANC was associated with a lower likelihood of neutrophil-specific autoantibody detection, more humoral abnormalities, and a shorter duration of neutropenia. These findings suggest two distinct patient clusters: one characterized by higher minimum ANC, more humoral abnormalities, and shorter neutropenia duration; and another with lower minimum ANC, fewer humoral abnormalities, more frequent autoantibody detection, and longer neutropenia duration.

Although the literature generally reports a slightly higher proportion of female patients, our study observed a predominance of male patients [5]. Consistent with previous studies, the IN group in our cohort also had a higher proportion of males [2]. Farruggia et al. highlighted that age at onset and absence of monocytosis in AIN patients, as well as age at onset in IN patients, significantly influenced neutropenia duration [13, 22]. Our findings further underscore the significant impact of humoral immune abnormalities, neutrophil-specific autoantibodies, and minimum ANC on the duration of early childhood transient neutropenia. Notably, 90% of patients in our cohort achieved remission within approximately 3 years, consistent with the literature reporting remission typically within two to three years after diagnosis [3, 5, 8].

Contrary to literature suggesting that IN patients are older at onset than AIN patients [2], our cohort showed the opposite: IN patients were younger at onset and remission and experienced a shorter duration of neutropenia. These findings do not support the hypothesis that IN patients are diagnosed later during the same disorder [2]. Consistent with previous studies [2], we confirmed that a lower ANC at the time of testing is associated with a higher likelihood of a positive neutrophil-specific autoantibody result. Our data indicate that an ANC above 0.5 × 10^9^/L at testing corresponds to a likelihood of less than 50% for a positive result, a finding that is particularly relevant for managing early childhood neutropenia in patients with negative autoantibodies. The higher ANC values observed in IN patients support the hypothesis that these patients may represent autoantibody-negative AIN cases with higher overall ANC [22], thus avoiding autoantibody detection and experiencing a shorter duration of neutropenia. Importantly, we included patients without autoantibody testing, as there were no significant differences in demographic or clinical characteristics between tested and untested IN patients, further supporting the notion that IN patients may often represent autoantibody-negative AIN cases [22].

Existing pediatric neutropenia studies offer limited direct comparators for the humoral immune findings reported here. Studies that included immunological assessment have largely focused on late-onset, long-lasting, or chronic neutropenia, reporting immune abnormalities including hypogammaglobulinemia, altered B-cell subsets, and previously unrecognized inborn errors of immunity in selected patients [31, 32]. These cohorts differ from the present study with respect to patient age, disease duration, referral pattern, and depth of immunological assessment, limiting direct quantitative comparison. To our knowledge, systematic humoral immune characterization at this level of granularity has not previously been reported in young children with transient autoimmune or idiopathic neutropenia, making our findings a novel descriptive baseline for this population.

Certain infections were more frequently recorded during the neutropenic phase, consistent with existing studies [3–5]. The observed increase in antibiotic-treated respiratory infections highlights the challenges in managing neutropenic patients, with a significant portion of this antibiotic use likely driven by concerns over neutropenia. This underscores a limitation of our retrospective study, as it complicates the assessment of the necessity of antibiotic treatment following a neutropenia diagnosis. Our analysis suggests a correlation between low minimum ANC and severe infections, as all patients with more than two severe infections had a minimum ANC ≤ 0.05 × 10^9^/L. Additionally, patients with lower minimum ANC were significantly more likely to be diagnosed with severe infections. However, these findings should be interpreted cautiously, as lower ANC may increase the likelihood of hospitalization, which we used to define severe infection. Infection documentation and admission thresholds likely varied across clinicians and over time, a common limitation of retrospective routine-care data. Further investigation into the relationship between ANC levels and infection risk is warranted.

As expected from the established link between antibody disorders such as CVID and autoimmune cytopenia [14–16, 18, 19], many patients in our cohort exhibited humoral abnormalities. In CVID, impaired B-cell differentiation and function are thought to promote hypogammaglobulinemia and the emergence of autoreactive B cell responses [16]. While selective IgA deficiency has been reported in approximately 3% of AIN and IN patients [22], we observed IgA and/or IgM deficiency in 53% of tested patients. This comparatively high proportion should be interpreted in the context of our study setting and design. Children with prolonged or unexplained neutropenia at our institution typically undergo a structured immunological evaluation according to established diagnostic pathways, allowing a relatively systematic assessment of humoral parameters in this clinical population. At the same time, elements of referral and ascertainment bias inherent to a tertiary-care setting cannot be fully excluded. Together with our inclusive definition, which captured isolated, mild, and potentially transient reductions in immunoglobulin levels in early childhood, this likely contributed to the observed frequency of abnormalities.

Accordingly, these findings should not be interpreted as population-level prevalences of persistent antibody deficiency, but rather as a detailed description of humoral immune findings observed in a selected cohort of young children with transient neutropenia managed in tertiary care. Deviations in B-cell subsets and reduced vaccine antibody responses in very young children may reflect age-dependent immune maturation and should be interpreted cautiously, as isolated abnormalities are insufficient to establish an inborn error of immunity (IEI) in the absence of persistence or additional supportive clinical or laboratory features. The observed frequency of humoral abnormalities reflects a combination of factors: the systematic and granular immunological assessment applied, the structured tertiary-care referral pathway, and the inclusive definitions used to capture the full spectrum of humoral deviations in this age group. Given that CVID and related antibody deficiencies cannot be diagnosed reliably before the age of four years, patients with persistent or evolving abnormalities require longitudinal follow-up.

Transient hypogammaglobulinemia of infancy (THI) may represent an associated condition in a subset of patients with reduced immunoglobulin levels in our cohort, though the boundaries between THI, physiological immune maturation, and AIN-associated humoral dysregulation remain poorly defined. A formal diagnosis of THI requires longitudinal documentation of low immunoglobulin concentrations followed by normalization to age-appropriate levels, which was not systematically available in this retrospective dataset. Whether normalization of immunoglobulin levels correlates temporally with remission of neutropenia therefore remains an open question. The potential co-occurrence and co-resolution of THI and transient neutropenia is biologically plausible and warrants dedicated investigation in prospective studies with standardized longitudinal immunological monitoring.

We propose that the humoral abnormalities observed in our cohort may reflect an underlying dysregulation in the adaptive immune system, potentially contributing to the etiology of transient neutropenia in early childhood. However, the consistent detection of neutrophil-specific autoantibodies in patients without humoral abnormalities challenges the notion of a direct causal role for these humoral abnormalities in AIN. The reason why patients without these abnormalities experience a longer duration of neutropenia remains unclear.

The broad search approach and the inclusion of 10 years of data enabled this study to include 92 patients and facilitated long-term follow-up. However, this approach also captured changes in medical practice over time, such as the reduced use of G-CSF in these patients. The involvement of multiple medical disciplines introduced variations in diagnostic algorithms, follow-up intervals, and patient care. Additionally, the retrospective design posed challenges, including missing data on the long-term progression of humoral abnormalities and the frequency and severity of infections. Vaccination histories were variably documented, limiting standardized assessment of vaccine responses in some patients. This design also carries inherent risks of selection, reporting, and confounding biases. Finally, IEI were not excluded by a single uniform test but were assessed longitudinally within routine tertiary immunology care, integrating clinical course, repeated laboratory assessments, and targeted investigations over time. Patients with features suggestive of congenital neutropenia, bone marrow failure, or syndromic immunodeficiency were excluded during cohort definition, and targeted genetic and/or bone marrow investigations were performed when clinically indicated. Referral and ascertainment bias inherent to a tertiary-care setting may have contributed to an over-representation of humoral immune abnormalities and could have influenced their apparent associations with clinical outcomes. However, the longitudinal and expert-driven nature of assessment mitigates the risk of misclassification and supports the clinical relevance of the observed findings. Given that neutropenia in preterm infancy may represent a distinct entity [29], we conducted a sensitivity analysis comparing preterm and term patients. Apart from differences in the context of initial neutropenia detection, likely reflecting surveillance practices, we observed no significant differences in key clinical outcomes or laboratory parameters, supporting the inclusion of preterm patients in the final cohort.

Despite these limitations, the study successfully uncovered new insights into humoral immune abnormalities in young children with transient neutropenia that may have been difficult to identify in a prospective study. These findings have important implications for future research and clinical management and warrant further investigation into immune dysregulation as a potential underlying cause of both humoral abnormalities and transient neutropenia in early childhood, ideally in prospective studies with predefined case report forms and standardized follow-up to allow systematic assessment of longitudinal immune trajectories and clinical outcomes. This should include systematic capture of autoimmune manifestations and detailed family history of autoimmunity or neutropenia. Additionally, exploring the relationship between low ANC and severe infections could provide important insights into the management and prognosis of these patients.

In conclusion, this study highlights the value of immunological testing focused on the humoral immune system in assessing the clinical course of transient neutropenia, predicting the likelihood of positive neutrophil-specific autoantibodies, and identifying inadequate vaccine responses requiring additional vaccine doses. We propose that dysregulation in the humoral immune system may contribute to the development of transient neutropenia in early childhood. By identifying two distinct patient clusters, this study provides a framework for clinicians to more accurately categorize patients, enabling more precise clinical assessments and potentially improving prognosis prediction.

## Supplementary Information

Below is the link to the electronic supplementary material.


Supplementary Material 1 (DOCX 644 KB)


## Data Availability

The datasets generated and analyzed during the current study are available from the corresponding author on reasonable request.
