# Supplementary material for: Humoral Immune Abnormalities in Transient Childhood Neutropenia: Insights From a 10-year Cohort Study in a Tertiary Center
Source: J Clin Immunol. 2026 Jun 18;46(1):85. doi: 10.1007/s10875-026-02042-w (PMC13526712; doi:10.1007/s10875-026-02042-w)
Supplement: Supplementary file 1 [file 10875_2026_2042_MOESM1_ESM.docx]

**Supplementary information**

**Article title: Humoral immune abnormalities in transient childhood neutropenia: Insights from a 10-Year cohort study in a tertiary center**

Emmanuele Schorn^1^, Maarja Soomann^1^, Seraina Prader^1^, Jana Pachlopnik Schmid^1^, Johannes Trück^1^

^1^ Division of Immunology and the Children’s Research Center, University Children's Hospital Zurich, University of Zurich, Zurich, Switzerland

Correspondence: Johannes Trück, MD, DPhil, Division of Immunology and Children’s Research Center, University Children’s Hospital Zurich, Lenggstrasse 30, 8008 Zürich, Switzerland (johannes.trueck@kispi.uzh.ch).

**Tables**

**Table S1: Definitions with corresponding explanations.**

| Term | Definition/Explanation |
| --- | --- |
| Autoimmune neutropenia (AIN) | Isolated neutropenia lasting more than 6 weeks with positive neutrophil-specific autoantibodies and no other identifiable cause(1) |
| Idiopathic neutropenia (IN) | IN was defined as neutropenia lasting more than 6 weeks, with either negative or no autoantibody testing and no other identifiable cause(2) |
| Inclusion criteria | 1) age 0 to 6 years, 2) transient neutropenia lasting for at least 6 weeks |
| Exclusion criteria | 1) an identifiable cause to their neutropenia other than autoimmunity (e.g., constitutional neutropenia, alloimmune neutropenia, neutropenia associated with myeloproliferative disorders, neutropenia related to acquired bone marrow failure, medication-induced neutropenia, and infection-associated neutropenia), 2) remission before 4 months of age |
| Late-onset neutropenia | Discovery of neutropenia after 36 months of age |
| Long-lasting neutropenia | Persistence of neutropenia for more than 36 months |
| Humoral abnormalities | 1) quantitative immunoglobulin deviations below age-adjusted reference ranges (IgG, IgA, IgM), and/or 2) abnormalities in B-cell subsets on flow cytometry interpreted against age-adjusted reference values, and/or 3) inadequate vaccine-specific antibody responses when assessable and interpretable based on documentation |
| Moderate humoral abnormalities | IgG deficiency and/or inadequate vaccine-specific antibody responses |
| Mild humoral abnormalities | IgA and/or IgM deficiency, IgG subclass deficiency, and/or abnormalities in B-cell differentiation, without moderate abnormalities |
| Severe infection | Documented infection requiring hospitalization |
| Mild infection | Documented infection not requiring hospitalization |

**Table S2: Search terms for patients with neutropenia.**

Search terms

PD Neutropenia AIN

AIN

autoimmune neutropenia

Autoimmun Neutropenie

Autoimmunneutropenie

Autoimmun-Neutropenie

Neutropenie autoimmune

Neutropenie, autoimmune

neutropenie

autoimmune

immun

auto

**Table S3: Neutropenia categories for sequential filtering process as seen in the flow diagram (Figure 1).**

| Neutropenia Group | Definition |
| --- | --- |
| No neutropenia |  |
| Denied general consent |  |
| > 6 years | Older than 6 years at onset of neutropenia |
| Not assessed | Not assessed for neutropenia at Children’s Hospital of Zurich |
| < 6 weeks neutropenia duration | Neutropenia duration less than 6 weeks |
| Infection associated neutropenia | Tuberculosis, HIV, CMV, Hepatitis B, several bacterial and viral infections |
| Constitutional Neutropenia | Severe congenital neutropenias, cyclic neutropenia, associated with various extra hematological signs, associated with congenital bone marrow failure, associated to metabolic disorders and nutritional deficiency as proposed by Fioredda et al (1) |
| Secondary autoimmune neutropenia | Neutropenia is secondary to another condition rather than being a primary disorder and is associated with other underlying conditions such as autoimmune diseases, malignancies, immunodeficiencies, viral infections, or drug reactions. It is more prevalent in adults and is often seen in conjunction with diseases like rheumatoid arthritis, Felty syndrome, systemic lupus erythematosus, scleroderma, large granular lymphocytic leukemia, chronic lymphocytic leukemia, Waldenström macroglobulinemia, Hodgkin lymphoma and thymoma (3) |
| Alloimmune Neutropenia | Neutropenia due to alloimmunization against neutrophil-specific antigens (4), autoantibodies are often detected in mother and child. Resolution of neutropenia in first 4 months after birth |

**Table S4: RStudio packages and corresponding citations.**

| RStudio packages | Citation |
| --- | --- |
| readxl | Wickham H, Bryan J (2023). _readxl: Read Excel Files_. R package version 1.4.3, <https://CRAN.R-project.org/package=readxl>. |
| dplyr | Wickham H, François R, Henry L, Müller K, Vaughan D (2023). _dplyr: A Grammar of Data Manipulation_. R package version 1.1.4, <https://CRAN.R-project.org/package=dplyr>. |
| lubridate | Garrett Grolemund, Hadley Wickham (2011). Dates and Times Made Easy with lubridate. Journal of Statistical Software, 40(3), 1-25. URL https://www.jstatsoft.org/v40/i03/. |
| ggplot2 | H. Wickham. ggplot2: Elegant Graphics for Data Analysis. Springer-Verlag New York, 2016. |
| tidyr | Wickham H, Vaughan D, Girlich M (2024). _tidyr: Tidy Messy Data_. R package version 1.3.1, <https://CRAN.R-project.org/package=tidyr>. |
| scales | Wickham H, Pedersen T, Seidel D (2023). _scales: Scale Functions for Visualization_. R package version 1.3.0, <https://CRAN.R-project.org/package=scales>. |
| hmisc | Harrell Jr F (2024). _Hmisc: Harrell Miscellaneous_. R package version 5.1-3, <https://CRAN.R-project.org/package=Hmisc>. |
| writexl | Ooms J (2024). _writexl: Export Data Frames to Excel 'xlsx' Format_. R package version 1.5.0, <https://CRAN.R-project.org/package=writexl>. |
| survival | Therneau T (2024). _A Package for Survival Analysis in R_. R package version 3.7-0, <https://CRAN.R-project.org/package=survival>.  Terry M. Therneau, Patricia M. Grambsch (2000). _Modeling Survival Data: Extending the Cox Model_. Springer, New York. ISBN 0-387-98784-3. |
| survminer | Kassambara A, Kosinski M, Biecek P (2021). _survminer: Drawing Survival Curves using 'ggplot2'_. R package version 0.4.9, <https://CRAN.R-project.org/package=survminer>. |
| ggpubr | Kassambara A (2023). _ggpubr: 'ggplot2' Based Publication Ready Plots_. R package version 0.6.0, <https://CRAN.R-project.org/package=ggpubr>. |
| broom | Robinson D, Hayes A, Couch S (2024). _broom: Convert Statistical Objects into Tidy Tibbles_. R package version 1.0.6, <https://CRAN.R-project.org/package=broom>. |
| boot | Angelo Canty and Brian Ripley (2022). boot: Bootstrap R (S-Plus) Functions. R package version 1.3-28.1.  Davison, A. C. & Hinkley, D. V. (1997) Bootstrap Methods and Their Applications. Cambridge University Press, Cambridge. ISBN 0-521-57391-2 |

**Table S5: Demographic, clinical and diagnostic characteristics of preterm and term born patients.**

|  | Preterm (n=22) | | Born at term (n=55) | | *p-value* |
| --- | --- | --- | --- | --- | --- |
|  | Number | (%) | Number | (%) |  |
| Sex (male) | 18 | (82) | 32 | (58) | *0.089* |
| Context of initial diagnosis of neutropenia |  |  |  |  | ***0.001*** |
| Incidental finding^*^ | 18 | (82) | 20 | (36) |  |
| Mild infection^#^ | 1 | (14) | 15 | (36) |  |
| Severe infection^$^ | 3 | (4) | 20 | (36) |  |
|  | Median | (Range) | Median | (Range) |  |
| Age at onset (months) | 4.4 | (0-29.5) | 7.9 | (1.0-31.5) | *0.071* |
| Age at remission (months) | 17.4 | (6.7-64.3) | 24.6 | (4.1-62.9) | *0.106* |
| Duration of neutropenia (months)^€^ | 10.3 | (4.6-46.6) | 16.1 | (1.3-50.1) |  |
| Lowest neutrophil value (x10^9^/L) | 0.2 | (0.02-0.7) | 0.16 | (0-0.88) | *0.498* |

*^*^full blood count (FBC) performed for reasons unrelated to infection; ^#^FBC due to infection, not hospitalized; ^$^FBC due to infection, hospitalized; ^€^in those who achieved remission*

**Table S6: Number of patients with mild and severe Infections before and during neutropenia.**

|  | Before neutropenia onset | | During neutropenia | |
| --- | --- | --- | --- | --- |
| Number of infections | Number of patients with mild infection^*^ | number of patients with severe infections^#^ | Number of patients with mild infections^*^ | Number of patients with severe infections^#^ |
| 0 | 67 | 55 | 23 | 56 |
| 1 | 8 | 31 | 24 | 19 |
| ≥2 | 17 | 6 | 45 | 17 |
| 2 | 8 | 2 | 7 | 8 |
| 3 | 2 | 4 | 18 | 4 |
| 4 | 3 | 0 | 10 | 1 |
| 5 | 2 | 0 | 4 | 0 |
| 6 | 0 | 0 | 2 | 2 |
| 7 | 1 | 0 | 1 | 0 |
| 8 | 1 | 0 | 0 | 0 |
| 9 | 0 | 0 | 2 | 0 |
| 10 | 0 | 0 | 0 | 1 |
| 11 | 0 | 0 | 0 | 0 |
| 12 | 0 | 0 | 0 | 1 |
| 13 | 0 | 0 | 1 | 0 |

*^*^Infection not requiring hospitalization; ^#^Infection requiring hospitalization*

**Table S7: Total number of infections among all patients before and during neutropenia, categorized by infection type.**

| Type of infection | before neutropenia | during neutropenia |
| --- | --- | --- |
| Respiratory infection treated with antibiotics | 2 | 21 |
| Respiratory infection without antibiotic treatment | 8 | 17 |
| Urinary tract infection | 6 | 8 |
| Enteritis | 2 | 8 |
| Pneumonia | 2 | 6 |
| Fever with hospitalization and intravenous antibiotics | 1 | 6 |
| Acute otitis media | 2 | 3 |
| Sepsis | 3 | 2 |
| Periorbital cellulitis / infraorbital phlegmon / preseptal cellulitis | 2 | 2 |
| Stomatitis | 0 | 2 |
| Line infection | 0 | 2 |
| Late onset sepsis | 3 | 1 |
| Conjunctivitis with intravenous antibiotics | 1 | 1 |
| Wound infection | 0 | 1 |
| Erysipelas | 0 | 1 |
| Mastitis | 0 | 1 |
| Paronychia | 0 | 1 |
| Omphalitis | 0 | 1 |
| Phlegmon | 0 | 1 |
| Furunculosis | 0 | 1 |
| Epstein-Barr Virus infection | 0 | 1 |
| Lymphadenitis | 6 | 0 |
| Cytomegalovirus infection | 2 | 0 |
| Dacrocystitis | 1 | 0 |
| Mastoiditis | 1 | 0 |
| Abscess | 1 | 0 |
| Retropharyngeal abscess | 1 | 0 |
| Perianal abscess | 1 | 0 |
| Early onset sepsis | 1 | 0 |
| Sum | 46 | 87 |

**Table S8: Demographic, clinical and diagnostic characteristics stratified by immunological testing.**

|  | Immunological testing  (n=68) | No immunological testing (n=24) | *p-value* |
| --- | --- | --- | --- |
|  | Number  (%) | Number (%) |  |
| Autoimmune Neutropenia | 45  (66) | 13 (54) | *0.423* |
| Idiopathic Neutropenia | 23  (34) | 11  (46) |  |
| Sex (male) | 38  (56) | 19 (79) | *0.076* |
| Preterm | 15  (22) | 7 (29) | *0.531* |
| Extremely preterm (<28 weeks) | 5  (7) | 3 (12.5) |  |
| Very preterm (28-31 weeks) | 3  (4) | 1 (4) |  |
| Moderate to late preterm (32-36 weeks) | 7  (10) | 3 (13) |  |
| Born at term | 43  (63) | 12 (50) |  |
| Unknown gestational age | 10  (15) | 5 (21) |  |
| Context of initial diagnosis of neutropenia |  |  |  |
| Incidental finding^*^ | 28  (41) | 16 (67) | *0.094* |
| Mild infection^#^ | 17  (25) | 4 (17) |  |
| Severe infection^$^ | 23  (34) | 4 (17) |  |
|  | Median (Range) | Median (Range) |  |
| Age at onset (months) | 7.8 (0-36.9) | 5.67 (0.7-12.8) | *0.233* |
| Age at remission (months) | 24.2 (4.1-68.8) | 16.3 (6.2-46.7) | *0.122* |
| Duration of neutropenia (months)^€^ | 15.6 (1.3-50.1) | 10.2 (2.8-37.1) |  |

*^*^full blood count (FBC) performed for reasons unrelated to infection; ^#^FBC due to infection, not hospitalized; ^$^FBC due to infection, hospitalized; ^€^in those who achieved remission*

**Table S9: Immunological abnormalities by diagnostic test in the autoimmune neutropenia and idiopathic neutropenia groups.**

|  | Autoimmune Neutropenia  (n=58) | | Idiopathic Neutropenia  (n=34) | |
| --- | --- | --- | --- | --- |
| Diagnostics | No. Tested | No. with positive test / abnormalities (%)^*^ | No. Tested | No. with positive test / abnormalities (%)^*^ |
| Autoantibody-testing | 58 | 58 (100) | 21 | 0 (0) |
| GIFT | 58 | 57 (98) | 18 | 0 (0) |
| GAT | 58 | 31 (54) | 18 | 0 (0) |
| ELISA (MAIGA) | 55 | 35 (64) | 17 | 0 (0) |
| Genetic testing | 11 | 0 (0) | 8 | 0 (0) |
| Bone marrow examination | 9 | 1 (0) | 1 | 0 (0) |
| Antibody testing^#^ |  |  |  |  |
| IgG | 46 | 7 (15) | 22 | 7 (32) |
| IgA and/or IgM | 46 | 18 (39) | 22 | 18 (82) |
| IgG subclasses | 14 | 3 (21) | 5 | 3 (60) |
| Vaccine antibodies | 46 | 11 (24) | 22 | 7 (32) |
| Flow cytometry |  |  |  |  |
| Total B, T, and NK cell count | 48 | 4 (8) | 22 | 0 (0) |
| T-cell subpopulation | 48 | 6 (13) | 21 | 1 (5) |
| B-cell subpopulation | 41 | 16 (39) | 20 | 8 (40) |

*^*^percentage of patients with abnormalities of all patients tested with the respective test; ^#^only decreased levels were considered abnormal; GIFT, granulocyte immunofluorescence test; GAT, granulocyte agglutination test; ELISA, enzyme-linked immunosorbent assay; MAIGA, monoclonal antibody immobilization of granulocyte-specific antigens; No., number*

**Table S10: Demographic, clinical and diagnostic characteristics by presence of humoral immune system abnormalities.**

|  | Any Abnormality (n=52) | No Abnormality (n=16) | *p-value* |
| --- | --- | --- | --- |
|  | Number (%) | Number (%) |  |
| Autoimmune Neutropenia | 29 (56) | 16 (100) | ***0.003*** |
| Idiopathic Neutropenia | 23  (44) | 0 (0) |  |
| Sex (male) | 31 (60) | 7 (44) | *0.407* |
| Preterm | 14 (27) | 1 (6) | *0.189* |
| Extremely preterm (<28 weeks) | 5 (9.6) | 0 (0) |  |
| Very preterm (28-31 weeks) | 3 (5.8) | 0 (0) |  |
| Moderate to late preterm (32-36 weeks) | 6 (11.5) | 1 (6) |  |
| Born at term | 31 (60) | 12 (75) |  |
| Unknown gestational age | 7 (14) | 3 (19) |  |
| Context of initial diagnosis of neutropenia |  |  |  |
| Incidental finding^*^ | 26 (50) | 2 (13) | ***0.023*** |
| Mild infection^#^ | 11 (21) | 6 (38) |  |
| Severe infection^$^ | 15 (29) | 8 (50) |  |
|  | Median (Range) | Median (Range) |  |
| Age at onset (months) | 6.2 (0-36.9) | 12.0 (5.3-34.2) | ***<0.001*** |
| Age at remission (months) | 19.9 (4.1-68.8) | 45.2 (18.5-62.9) | ***<0.001*** |
| Duration of neutropenia (months)^€^ | 12.1 (1.3-50.1) | 28.5 (13.2-40.4) |  |

*^*^full blood count (FBC) performed for reasons unrelated to infection; ^#^FBC due to infection, not hospitalized; ^$^FBC due to infection, hospitalized; ^€^in those who achieved remission*

**Table S11: Demographic, clinical and diagnostic characteristics of autoimmune and idiopathic neutropenia patients.**

|  | Autoimmune Neutropenia  (n=58) | | Idiopathic Neutropenia  (n=34) | | *p-value* |
| --- | --- | --- | --- | --- | --- |
|  | Number | (%) | Number | (%) |  |
| Sex (male) | 34 | (41) | 23 | (68) | *0.523* |
| Preterm | 10 | (17) | 12 | (35) | *0.130* |
| Extremely preterm (<28 weeks) | 4 | (7) | 4 | (12) |  |
| Very preterm (28-31 weeks) | 0 | (0) | 4 | (12) |  |
| Moderate to late preterm (32-36 weeks) | 6 | (10) | 4 | (12) |  |
| Born at term | 37 | (64) | 18 | (53) |  |
| Unknown gestational age | 11 | (19) | 4 | (12) |  |
| Context of initial diagnosis of neutropenia |  |  |  |  | ***0.002*** |
| Incidental finding^*^ | 20 | (35) | 24 | (71) |  |
| Mild infection^#^ | 15 | (26) | 6 | (18) |  |
| Severe infection^$^ | 23 | (40) | 4 | (12) |  |
|  | Median | (Range) | Median | (Range) |  |
| Age at onset (months) | 9.0 | (0.7-36.9) | 4.3 | (0-27.3) | ***0.001*** |
| Age at remission (months) | 29.2 | (6.1-68.8) | 16.3 | (4.1-47.2) | ***0.005*** |
| Duration of neutropenia (months)^€^ | 17.4 | (1.3-50.1) | 9.6 | (2.8-46.0) |  |

*^*^full blood count (FBC) performed for reasons unrelated to infection; ^#^FBC due to infection, not hospitalized; ^$^FBC due to infection, hospitalized; ^€^in those who achieved remission*

**Table S12: Demographic, clinical and diagnostic characteristics of idiopathic neutropenia patients with and without autoantibody testing.**

|  | IN Patients tested  (n=21) | | IN Patients not tested  (n=13) | | *p-value* |
| --- | --- | --- | --- | --- | --- |
|  | Number | (%) | Number | (%) |  |
| Sex (male) | 15 | (71) | 8 | (62) | *0.709* |
| Preterm | 8 | (38) | 4 | (31) | *1.000* |
| Extremely preterm (<28 weeks) | 2 | (10) | 2 | (15) |  |
| Very preterm (28-31 weeks) | 3 | (14) | 1 | (8) |  |
| Moderate to late moderate preterm (32-36 weeks) | 3 | (14) | 1 | (8) |  |
| Born at term | 11 | (52) | 7 | (54) |  |
| Unknown gestational age | 2 | (10) | 2 | (15) |  |
| Context of the initial diagnosis of neutropenia |  |  |  |  | *0.539* |
| Incidental finding^*^ | 14 | (67) | 10 | (77) |  |
| Mild infection^#^ | 5 | (24) | 1 | (8) |  |
| Severe infection^$^ | 2 | (10) | 2 | (15) |  |
|  | Median | (Range) | Median | (Range) |  |
| Age at onset (months) | 3.38 | (0-27.3) | 5.3 | (1.2-11.7) | *0.625* |
| Age at remission (months) | 15.8 | (4.1-41.1) | 16.4 | (6.2-47.2) | *0.550* |
| Duration of neutropenia (months) | 8.79 | (2.8-32.6) | 10.2 | (4.2-46) |  |

*^*^full blood count (FBC) performed for reasons unrelated to infection; ^#^FBC due to infection, not hospitalized; ^$^FBC due to infection, hospitalized; ^€^in those who achieved remission; IN, idiopathic neutropenia*

**Table S13: Demographic, clinical and diagnostic characteristics of patients with minimum absolute neutrophil count values ≥0.25 × 10^9^/L and <0.25 × 10^9^/L.**

|  | Minimum ANC  <0.25 × 10^9^/L  (n=53) | | Minimum ANC  ≥ 0.25 × 10^9^/L (n=39) | | *p-value* |
| --- | --- | --- | --- | --- | --- |
|  | Number | (%) | Number | (%) |  |
| Autoimmune Neutropenia | 44 | (83) | 14 | (36) | ***<0.001*** |
| Idiopathic Neutropenia | 9 | (17) | 25 | (64) |  |
| Sex (male) | 31 | (59) | 26 | (67) | *0.561* |
| Preterm | 11 | (21) | 11 | (28) | *0.690* |
| Extremely preterm (<28 weeks) | 4 | (8) | 4 | (10) |  |
| Very preterm (28-31 weeks) | 0 | (0) | 4 | (10) |  |
| Moderate to late preterm (32-36 weeks) | 7 | (13) | 3 | (8) |  |
| Born at term | 32 | (60) | 23 | (59) |  |
| Unknown gestational age | 10 | (19) | 5 | (13) |  |
| Context of the initial diagnosis of neutropenia |  |  |  |  | ***0.007*** |
| Incidental finding^*^ | 19 | (36) | 25 | (64) |  |
| Mild infection^#^ | 12 | (23) | 9 | (23) |  |
| Severe infection^$^ | 22 | (42) | 5 | (13) |  |
|  | Median | (Range) | Median | (Range) |  |
| Age at onset (months) | 9.5 | (0.7-36.9) | 2.9 | (0-27.3) | ***<0.001*** |
| Age at remission (months) | 30.4 | (6.1-68.8) | 14.9 | (4.1-60.9) | ***<0.001*** |
| Duration of neutropenia (months)^€^ | 18.3 | (1.3-46.6) | 9.2 | (2.5-50.1) |  |

*^*^full blood count (FBC) performed for reasons unrelated to infection; ^#^FBC due to infection, not hospitalized; ^$^FBC due to infection, hospitalized; ^€^in those who achieved remission; ANC, absolute neutrophil count*

**Figures**

**Figure S1: Time to remission of neutropenia.** Probability of remission including 95% confidence interval is shown for idiopathic neutropenia patients with and without neutrophil-specific autoantibody testing. Dashed lines indicate the median time to remission in the two subgroups.

**Figure S2: Time to remission of neutropenia.** Probability of remission including 95% confidence interval is shown for patients with moderate (minimum absolute neutrophil count (ANC) 0.5-1 × 10^9^/L) and severe neutropenia (minimum ANC < 0.5 × 10^9^/L). Dashed lines indicate the median time to remission in the two subgroups.

**Figure S3: Absolute neutrophil count values up to 3 months after remission of neutropenia.** The different subgroups moderate, mild, and no humoral abnormalities, as well as the group of patients without additional immunological diagnostics are indicated with different colors. A smooth curve shows the general course the absolute neutrophil count.

**References**

1.	Fioredda F, Onofrillo D, Farruggia P, Barone A, Veltroni M, Notarangelo LD, et al. Diagnosis and management of neutropenia in children: The approach of the Study Group on Neutropenia and Marrow Failure Syndromes of the Pediatric Italian Hemato-Oncology Association (Associazione Italiana Emato-Oncologia Pediatrica - AIEOP). Pediatr Blood Cancer. 2022 Jun;69(6):e29599.

2.	Farruggia P, Fioredda F, Puccio G, Onofrillo D, Russo G, Barone A, et al. Idiopathic neutropenia of infancy: Data from the Italian Neutropenia Registry. Am J Hematol. 2019 Feb;94(2):216–22.

3.	Fioredda F, Dufour C, Höglund P, Papadaki HA, Palmblad J. Autoimmune Neutropenias: Update on Clinical and Biological Features in Children and Adults. HemaSphere [Internet]. 2023 Jan [cited 2024 Apr 30];7(1). Available from: https://www.ncbi.nlm.nih.gov/pmc/articles/PMC9771305/

4.	Farruggia P, Dufour C. Diagnosis and management of primary autoimmune neutropenia in children: insights for clinicians. Ther Adv Hematol. 2015 Feb;6(1):15–24.
